# Pore morphology of bijel-templated materials promotes migration and downregulates αSMA expression in human fibroblasts

**DOI:** 10.3389/fbioe.2025.1709302

**Published:** 2025-12-16

**Authors:** Alyse R. Gonthier, Elliot L. Botvinick, Ali Mohraz

**Affiliations:** 1 Department of Materials Science and Engineering, University of California, Irvine, CA, United States; 2 Department of Biomedical Engineering, University of California, Irvine, CA, United States; 3 Department of Surgery, University of California, Irvine, CA, United States; 4 Beckman Laser Institute, University of California, Irvine, CA, United States; 5 Edwards Lifesciences Foundation Cardiovascular Innovation and Research Center, University of California, Irvine, CA, United States; 6 Department of Chemical and Biomolecular Engineering, University of California, Irvine, CA, United States

**Keywords:** biomaterial, bijel, microstructure, migration, phenotype, fibroblast

## Abstract

The efficacy of an implanted biomaterial is dependent on its ability to mitigate the foreign body response (FBR). Consequently, advancements in biomaterials design often focus on their immunomodulatory capability. Porous scaffolds have proven especially useful due to their capacity for cell infiltration and biomimicry of native tissues. Specifically, substrates with distinct microstructures have been shown to reduce fibrotic production and increase vascularization *in vivo*. Investigation of the direct relationship between FBR-implicated cell behavior and these materials, known as bicontinuous interfacially jammed emulsion gels (bijel)-templated materials (BTMs), has not yet been performed experimentally. In this study, we examine the influence of BTMs on the characteristics of human fibroblasts and assess the validity of existing computational results. The BTM, with its uniform pore size and negative Gaussian surface curvatures, is compared to the particle-templated material (PTM), which has constricting pore networks and variable surface curvature. Fibroblasts seeded into BTMs had less circular shapes, larger areas, increased motility, and reduced inflammation compared to cells seeded into PTMs. The specific behavior of cells within the PTM suggests that the reduction in migratory capability results from high local surface curvature. This corroborates previous computational work which predicted similar differences in cell shape and migration, as well as the influence of local curvature. These new experimental results provide key insights into the interaction between fibroblasts and biomaterial microstructure, prompting further investigation into the mechanisms behind these relationships.

## 1 Introduction

The native biological response to implanted materials continues to limit the benefits of biomedical devices (Anderson et al., 2008; Ward, 2008; Mariani et al., 2019). While this reaction, called the foreign body response, is evolutionarily beneficial, modern medical advancements often seek to artificially manipulate it in order to maximize device efficacy (Veiseh et al., 2015; Chu et al., 2020; Kharbikar et al., 2021). This manipulation is achievable through many routes, including surface coatings (Xiao et al., 2023), biodegradable components (Chen et al., 2014), and drug delivery mechanisms (Fenton et al., 2018). Control solely by microstructural means, however, is particularly advantageous because it avoids concerns of chemical leeching or non-specific delivery of active molecules (Leong et al., 2013; Zhou et al., 2021). Physical control of the foreign body response is possible thanks to known relationships between cells, their reactions to physical biomaterial properties, and how those reactions contribute to the immune response. Some cell types have well-established relationships: macrophages reliably polarize to a pro-healing phenotype if they are forced to elongate (McWhorter et al., 2013), while fibroblasts exhibit more inflammation on surfaces with less roughness (Kim et al., 2015; Huang et al., 2022). Understanding the relationships between cells and more complex substrate features is of great interest in pursuit of improving the lifetime and efficacy of implantable devices. While *in vivo* studies often derive sufficient knowledge of the immune response to catapult these materials towards clinical use, the more broadly impactful, underlying fundamental insights are not always evaluated. This is in part due to the challenges associated with elucidating specific biological information from multi-faceted cell-material interactions. Computational modeling is a useful supplement to this end, as it allows initial investigation which can motivate *in vitro* study at low cost and with minimal resources.

**FIGURE 1:**
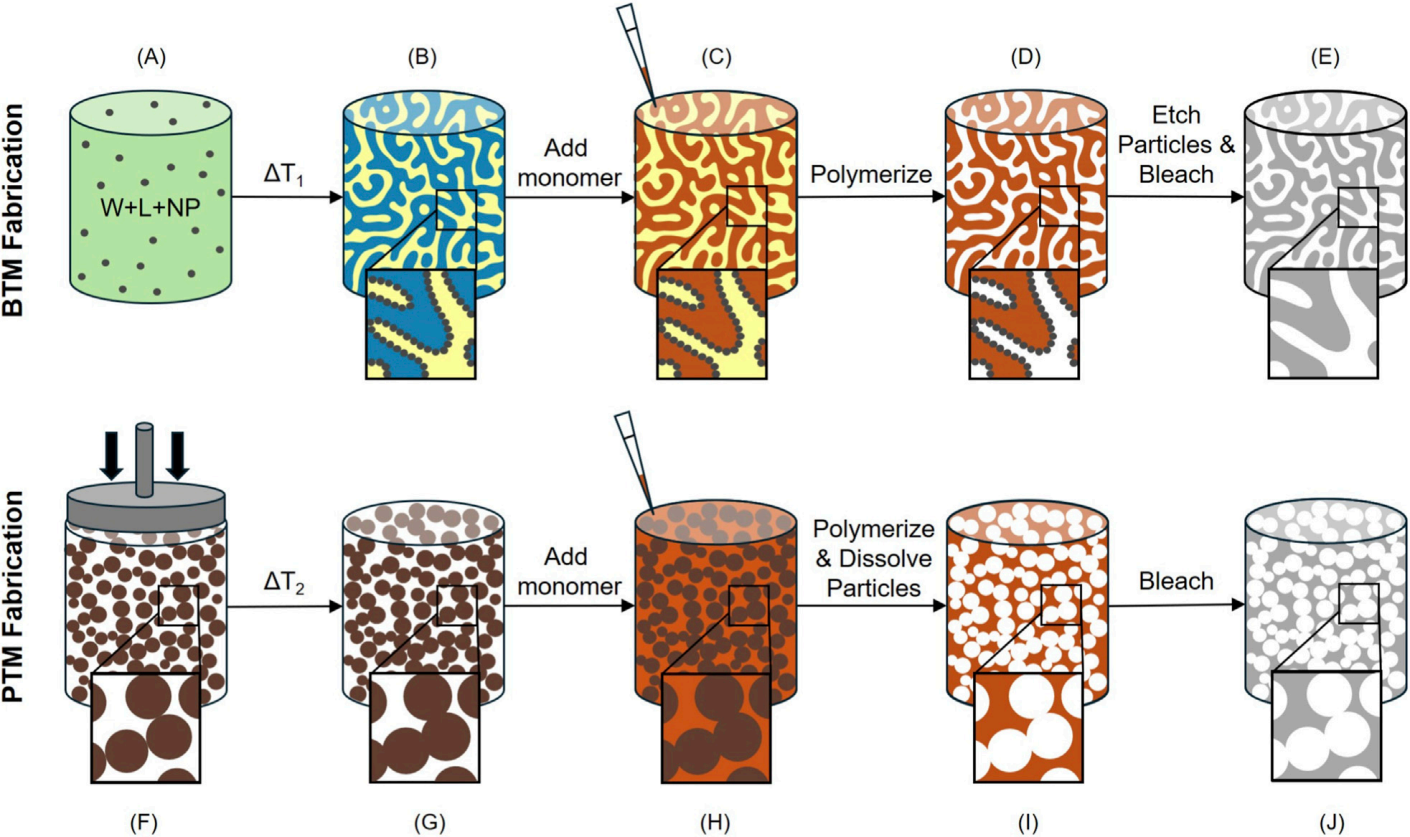
Fabrication schematic for BTMs and PTMs. BTM fabrication is described in detail in Section 2.1.1 and is summarized in the depicted steps: **(A)** water, lutidine, and nanoparticle mixture is heated to achieve **(B)** a bijel structure, with nanoparticles at the interface. Then, **(C)** a monomer precursor solution is added and selectively partitions into one fluid phase. The sample is then polymerized, creating **(D)** a solid BTM with nanoparticles remaining at the interface. Finally, the sample is etched to remove the nanoparticles and bleached to remove excess dye, resulting in **(E)** a completed BTM. PTM Fabrication is described in detail in Section 2.1.2 and is summarized in the depicted steps: **(F)** microparticles are compacted into a cylinder and subsequently heated, causing **(G)** the microparticles to fuse together. Then, **(H)** a monomer precursor solution is added to fill in the remaining void phase. **(I)** The initial microparticles are dissolved away, and the final structure is bleached to remove excess dye, resulting in **(J)** a completed PTM.

**FIGURE 2:**
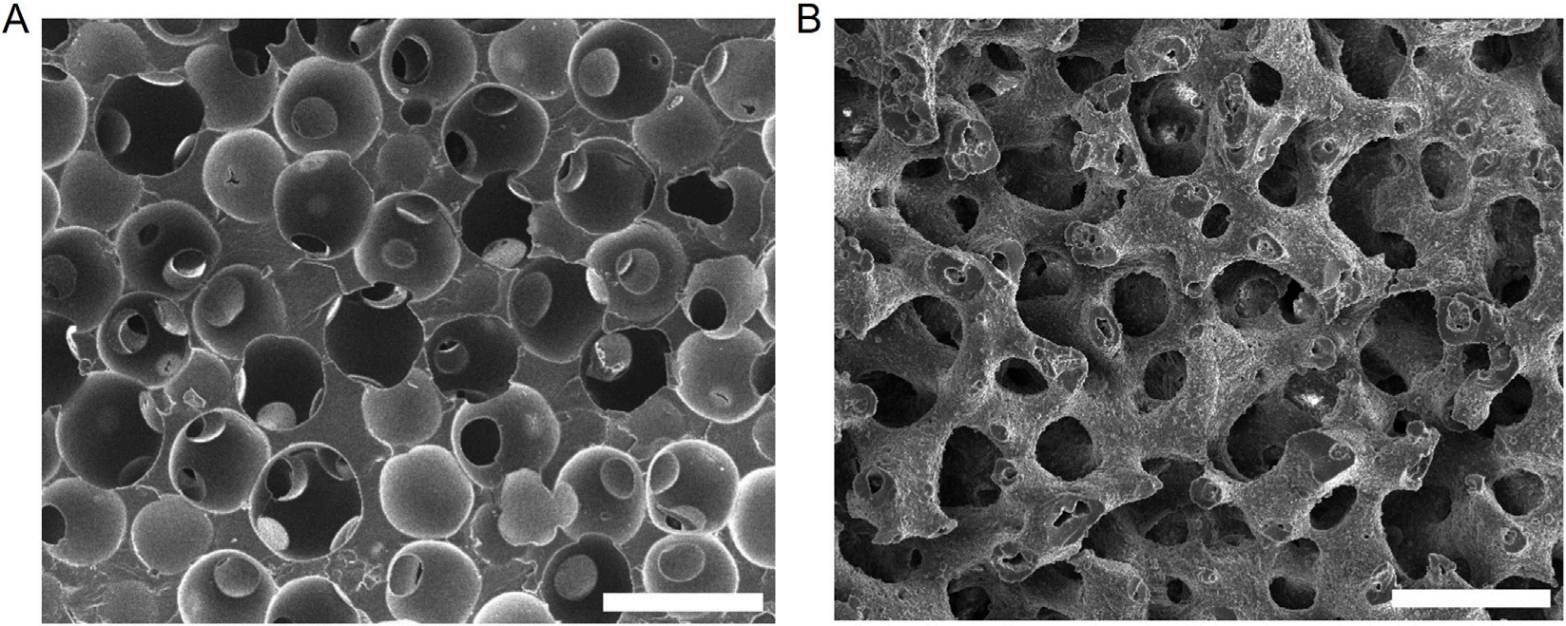
Porous substrates visualized via scanning electron microscopy. Poly (ethylene glycol) diacrylate samples of a **(A)** PTM and **(B)** BTM are pictured. Scale bar, 100 µm.

One recent computational study by our group examined porous biomaterials and found that their curvature landscapes dictated the shape and migration behavior of generic cells (Gonthier et al., 2023). That study was motivated by a preceding *in vivo* investigation which demonstrated significant differences in the overall foreign body response between the tested scaffolds (Thorson et al., 2019). These studies examined two materials: the bicontinuous interfacially jammed emulsion gel (bijel)-templated material (BTM) (Stratford et al., 2005; Thorson et al., 2018) and the particle-templated material (PTM) (Bhrany et al., 2013; Sussman et al., 2014). The substrates were chemically equivalent and had the same pore size, thus differing only in pore morphology. The PTM is made simply by fusing together a volume of spherical particles and subsequently inverting it to create a material with spherical, interconnected pores (Thorson et al., 2018; Calleros et al., 2020) (Figures 1, 2A). Materials like the PTM have been shown previously to have a positive effect on foreign body response compared to non-porous materials and are currently being investigated by other groups for clinical applications (Sussman et al., 2014; Zhen et al., 2025). Notably, the pore throats at the interconnects are only 1/3 the diameter of the spheres (Sussman et al., 2014; Teng et al., 2014; Chan et al., 2022). In comparison, the BTM boasts continuous, relatively uniform pore size throughout the substrate volume, owed to the thermodynamic process by which its parent bijel is created. The bijel results from the spinodal decomposition of two partially miscible fluids with neutrally wetting particles at their interface (Lee and Mohraz, 2010; Lee et al., 2013; Witt et al., 2013). At a characteristic length, which can be controlled via the particle volume fraction, the interfacially-adsorbed particles jam and prevent any further demixing (Clegg et al., 2007). The resultant bijel structure has a relatively uniform pore diameter and a predominance of negative Gaussian (saddle-like) curvature (Casey, 1996) along its internal surfaces. By infiltrating one of the fluid phases with a monomer solution and photopolymerizing it, the unique morphological characteristics of the bijel template are retained in the resultant porous BTM (Figures 1, 2B).

When implanted in rodents, the BTM caused a comparatively positive foreign body response, characterized by less fibrosis around the implant, a higher ratio of pro-healing (M2) macrophages, and better cell infiltration into the implant depth, which are associated with better biomaterial integration and demonstrate translational relevance (Hannan et al., 2018). It is not known, however, which specific cell types are directly affected by the implant microstructure and in what way, in contrast to cell types which may only experience downstream effects or a combination thereof. The existing computational work suggests that generic cells in BTMs will migrate more and take on more complex cell shapes than cells in PTMs (Winkler et al., 2019; Gonthier et al., 2023). One required characteristic of the generic cell type used in the model is locomotion via actin-ratcheting. Fibroblasts are the ideal initial subject of investigation, then, because they satisfy the actin-ratcheting condition and are also a key player in the foreign body response. We specifically chose normal human dermal fibroblasts (NHDFs) for this study, because they are known to be sensitive to microenvironmental cues, including substrate stiffness and micropatterning (Figallo et al., 2007; Jiang et al., 2011; D’Urso et al., 2025). This study thus aims to provide the initial investigation into the nature of fibroblasts’ direct interaction with BTMs and PTMs, which will serve both to assess the experimental validity of existing computational predictions, as well as to guide future and cumulatively comprehensive results for other FBR-implicated cell types.

To study fibroblasts *in vitro*, we chose a pore size of 80 μm, which corresponds to a PTM pore throat diameter of 20–25 µm. Fibroblasts can extend for 100s of microns in length (Tai et al., 2022) and have nuclei on the order of 10 µm (Poignant et al., 2022). Thus, 80 µm substrates allowed us to investigate the effects of pore morphology while avoiding any purely physical hindrance which would prevent fibroblasts from interrogating the structures. We hypothesized that the fibroblasts, as main effectors in the foreign body response, would exhibit differences in shape and migration in response to the disparate curvatures of the two substrate morphologies. We suggest, then, that any subsequent differences in phenotype that arise are as a direct result of these morphological changes, providing new evidence into the cascade which caused an overall anti-inflammatory response *in vivo*. Rather than attempting to replicate *in vivo* conditions, this study serves as a mechanistic bridge between prior *in vivo* and computational work, demonstrating that fibroblasts themselves respond directly to substrate architecture. To achieve this examination, fibroblasts were seeded directly into BTM and PTM substrates made of the same polymer. Cells were evaluated for their shape, size, motility, and phenotype to directly measure the effect of pore morphology on metrics relevant to fibroblast function.

## 2 Materials and methods

### 2.1 Preparation of substrates

Fabrication schematics for both the BTM and the PTM are presented in Figure 1.

#### 2.1.1 Bijel-templated material (BTM)

BTMs were formed following existing protocols (Thorson et al., 2018; 2019). Fluorescent silica nanoparticles were synthesized from Rhodamine B (Sigma-Aldrich) conjugated (3-aminopropyl) triethoxysilane (APTES, TCI America), tetraethyl orthosilicate (TEOS, Sigma-Aldrich), ethanol, and strong ammonium hydroxide solution (Fisher Chemical), using the Stöber synthesis method (van Blaaderen and Vrij, 1992). The formed particles were washed with deionized water via centrifugation three times (VWR Clinical 200). The particles were spread out on a glass Petri dish and dried in a vacuum oven (135 °C). The particles were continually dried until they reached a state of neutral wetting with respect to 2,6-lutidine and water. This neutral wetting property causes the particles to rest at the interface of the water-rich and lutidine-rich phases of the binary fluid mixture (White et al., 2011; Huang et al., 2017). In our protocol, particles were first ultrasonically dispersed (Branson Sonifier 250, Emerson) in water at a volume fraction of 2.5% v/v. Bijels were then formed by mixing the appropriate amount of the aqueous nanoparticle suspension with 2,6-lutidine to create a binary fluid mixture at its critical composition (lutidine mole fraction x_L_ = 0.064), pipetting all of the mixture into a glass, cylindrical (5 mm diameter) holder, and rapidly heating it in a microwave for 30 s to initiate spinodal decomposition of the binary fluid mixture. The vessel was immediately capped with aluminum foil to prevent evaporation. The samples were placed in an oven for 5 min at 70 °C. A monomer solution of poly (ethylene glycol) diacrylate (M_n_: 258 g/mol, Sigma-Aldrich) with 1% v/v photoinitiator (Darocur 1173^©^, Ciba Specialty Chemicals) was gently pipetted on the top surface of the bijel. The vessel was quickly recapped and placed in the 70 °C oven for 4 h. Once the polymeric solution had diffused throughout the lutidine-rich phase, the vessel was photopolymerized for 2 min (UV light, 100 W/cm^2^, λ = 320–390 nm). The resultant structure is a microporous polymeric substrate at a final composition of 36.2% v/v PEGDA. The BTM samples were washed in isopropyl alcohol and water and sliced into disks approximately 1 mm thick. To remove the silica particles from the internal surfaces of the substrate, samples were etched with hydrofluoric acid (HF) for 6 h. Samples were washed in an excess of water for a minimum of ten cycles to ensure full removal of the HF solution. Any residual Rhodamine B still adhered to the substrates was degraded by soaking in a sodium persulfate solution for at least 1 h (0.2 g/mL in water, Sigma-Aldrich) and irradiating with UV-light (100 W/cm^2^, λ = 320–390 nm) in 10-min intervals until the samples were optically white (Chen et al., 2012).

#### 2.1.2 Particle-templated material (PTM)

PTMs were formed based on previous methods (Sussman et al., 2014; Thorson et al., 2019). A cylindrical vessel, 5 mm in diameter, was loaded with poly (methyl methacrylate) particles (PMMA, diameter = 75–90 μm, Cospheric). To help ensure a randomly close-packed arrangement of the particles, a 16-gauge needle was used to gently agitate the particles throughout the vessel volume, minimizing clumping or significant gaps. The samples were compacted into a particle bed using a syringe plunger (5 mm diameter). The samples were placed under vacuum for 5 min, before heating in an oven at 180 °C for 2 h to reach a particle-particle connection diameter of approximately 25 µm by fusing particles with their neighbors at the elevated temperature. After cooling to room temperature for 30 min, the fused particle bed was removed from the cylindrical vessel. A monomer solution containing 36.2% v/v PEGDA with 1% photoinitiator in 2,6-lutidine was prepared (see Section 2.1.1.) with the addition of 0.1 mg/mL Rhodamine B. This solution was pipetted onto the fused particles and photopolymerized as described previously (Section 2.1.1). Fully solidified samples were cut into disks approximately 1 mm thick and soaked overnight in ethyl acetate (Sigma-Aldrich) to dissolve the PMMA particles. Samples were washed multiple times in ethyl acetate and ethanol, before washing in ethanol-water mixtures of 50% and 70% (v/v). The disks were then soaked in a sodium persulfate solution (0.2 g/mL) for 1 h and UV-irradiated. Finished samples were washed at least 3 times in water, with sonication. The inclusion of Rhodamine B in the PTM monomer solution was designed to best match the BTM and PTM chemical states and post-processing steps, to the extent possible.

#### 2.1.3 Substrate preparation for cell culture

Completed BTMs and PTMs were prepared for cell culture by a series of 5 wash steps in sterile 70% ethanol, with 5 min of sonication at each step. Samples were soaked in 70% ethanol overnight prior to introduction to a biosafety hood. The substrates were washed 3 times, with vigorous mixing, in sterile Dulbecco’s phosphate-buffered saline (PBS, +MgCl_2_ +CaCl_2_, Gibco) in a sterile cell culture hood. Substrates were then washed in supplemented Dulbecco’s Modified Eagle Medium (DMEM, Gibco), containing 1% v/v penicillin-streptomycin (P/S, Gibco) and 10% v/v fetal bovine serum (FBS, Sigma-Aldrich). The washing media was removed and each substrate was resubmerged in fresh culture media in individual plate wells. These samples were incubated (5% CO_2_, 37 °C) for 30 min preceding cell introduction.

### 2.2 Cell culture

#### 2.2.1 Fibroblasts

Normal human dermal fibroblasts (NHDFs, adult, Lonza) were cultured in T-75 flasks with supplemented cell culture media (see Section 2.1.3). Cells were passaged at 70%–90% confluency, up to passage 15 per vendor recommendation and in line with existing studies (Kamiya et al., 2021; D’Urso et al., 2025). NHDFs were lifted using 0.05% trypsin (5 min, incubated). Cells were centrifuged at 300 g for 5 min prior to resuspension and plating (Eppendorf 5910 Ri). NHDFs were plated in glass-bottom 24-well plates (100,000 cells/well counted by hemocytometer).

#### 2.2.2 Cell viability

BTM and PTM samples were added to each well, separated from direct cell contact by transwell constructs in order to study the effects of any molecules leaching from the substrates. Cells were stained for survival (Live/Dead cell imaging kit, ThermoFisher) and nuclei (NucBlue, Hoechst 33342, Invitrogen) according to manufacturer instructions after 48 h. Confocal images were taken (see Section 2.4.1) for total viability analysis. At least 1,000 total cells were measured per substrate type, from at least 3 separate samples. Separately, substrate samples were directly seeded with cells (see Section 2.3.1) and stained to provide visual confirmation of live cell presence within the substrates.

### 2.3 Cell-substrate studies

#### 2.3.1 Cell seeding in substrates

First, NHDFs were lifted, centrifuged (see Section 2.2.1), and resuspended at high concentration (∼6 × 10^6^ cells/mL). A volume of 8–15 µL was added to each substrate or well, seeding ∼80,000 cells each by pipetting small fractions of the total injection volume at various locations across the substrate surface. All BTMs, PTMs, and controls were seeded concurrently and with the same batch of cells, which had the same lot and passage number. Excess media was minimized to prevent significant spread of the liquid throughout the well. This helps to prevent cells from readily flowing out of the substrate and on to the glass. After incubation for 1 h to allow initial attachment, substrates were carefully transferred to new wells. An appropriate volume of media was added to each new well (e.g., 500 μL for a 24-well plate). Samples were further processed after incubation for 48 h.

Cells were also seeded on glass, without a substrate, to provide a control for cell health and behavior. By plating on planar, non-porous glass, any challenges arising from cell culturing methodology could be identified. The substrates of interest in this study are inherently three-dimensional, which is known to induce significantly different cell behavior than two-dimensional substrates (Li and Kilian, 2015; Duval et al., 2017). Given that the purpose of this investigation is to compare cell behavior in BTMs to cell behavior in PTMs, we emphasize only that comparison, and do not seek to characterize cell behavior in an absolute manner in comparison to an alternate two-dimensional control.

#### 2.3.2 Migration

Samples for visualizing cell migration were prepared similarly to those described in Section 2.3.1., with some additional steps. Prior to lifting the cells, two drops of NucBlue (Hoechst 33342, Invitrogen) were added per mL of culture media, per manufacturer instruction, and the cells were incubated for 20 min. Cells were subsequently lifted and seeded into substrates. After 4 h of initial attachment, samples were inverted and moved to a 35 mm glass-bottom Petri dish (Alkali Scientific). The volume of excess media in this dish was optimized such that samples remained fully submerged throughout the duration of the study, but did not have enough room to be significantly jostled from their original position. This is necessary to allow the visualization of multiple regions over time per sample, without disrupting the sample positions upon x-y movement of the microscope stage.

Only the single nuclear stain was utilized for migration studies, in an effort to minimize the potential side effects and phototoxicity associated with live cell imaging (Magidson and Khodjakov, 2013; Daddysman et al., 2014; Reyes Lua et al., 2021). Migration studies were optimized for minimal loss due to procedure, while the fixed cell shape studies were performed separately. We note that these materials, while three-dimensional in nature, are isotropic. Thus any 2D compression for the presentation of data will not result in any loss of information which would differentiate the two substrates.

Migrating cells were quantified according to their maximum distance from their original location (radius from origin, **r**), as well as their total area of migration, which is defined as the minimum size bounding box which contains the entire migratory space. Let us consider 2 cells which have the same maximum radius from origin. Cell A only travels one path from the origin to a point at a distance R from origin. Cell B travels the same initial path as Cell A, but then migrates in the opposite direction, passing back through the origin, and arriving at a new point that is also a distance R from the origin. These 2 cells have the same maximum radius from origin, even though Cell B traveled much more than Cell A. The migration area metric accounts for this discrepancy, and would assign Cell B a migration area four times larger than the migration area for Cell A. However, let us also consider Cell C which migrates a distance 2R from its origin in one direction. Cell B and Cell C have the same migration area, despite the fact that Cell C traveled much farther away from its initial position. Together, these two metrics provide a contextualized look at not only overall migratory behavior, but also the tendency of cells to be trapped in one region.

#### 2.3.3 Fluorescent staining for microscopy

*In situ* visualization was performed via actin filament (F-actin) and nuclei staining. Samples were moved to new wells and rinsed gently with sterile PBS. Cells were fixed for 15 min (Z-Fix, Cancer Diagnostics), prior to permeabilization for 12 min (0.1% Triton-X 100, Thermo Scientific) and blocking for 30 min (3% bovine serum albumin, ThermoFisher). Samples were washed in PBS three times between each step listed. The staining solution, applied for 30 min, contained AlexaFluor488 Phalloidin (1:800, Invitrogen) and 4′,6-diamidino-2-phenylindole, dihydrochloride (DAPI, 1 μg/mL, Invitrogen). Completed samples were adjusted such that the injected surface was closest to the glass.

#### 2.3.4 Cell retrieval and flow cytometry

Fibroblasts were retrieved from the substrates by incubating with 0.05% trypsin for 5 min. The contents of each well were mixed via pipetting multiple times, to dislodge as many cells as possible. The cell suspension was transferred to a microcentrifuge tube and spun at 400 g for 5 min. Each subsequent step was followed by 3 washing steps, via centrifugation in flow buffer (PBS with 1% FBS). Samples were gently resuspended in 400 µL of flow buffer and an equal volume of Z-Fix and left for 15 min. A combined permeabilization and blocking step followed, via a solution containing 0.1% Triton-X 100% and 3% FBS, for 12 min. Primary mouse anti-α-SMA monoclonal antibody (1:200, Invitrogen), was added for 45 min. Then, secondary antibody, goat anti-mouse Cy5 (1:200, Invitrogen), and actin stain AlexaFluor488 Phalloidin (1:800) were added for 30 min. Samples for phenotype controls were seeded onto plain glass and cultured for 24 h in media containing a reduced 5% FBS. Cells were exposed to either 0 ng/mL TGF-β or 15 ng/mL TGF-β (R&D Systems) for 24 h. These cells were then subjected to the staining procedure described above. All samples were washed, covered, and stored in flow buffer.

Flow cytometry was performed on a 4-Laser NovoCyte Quanteon Flow Cytometer System (Agilent). Proper gating was determined by first measuring unstained, minimally processed cells cultured on glass. The apparent gates for that cell population were then compared to cells stained for their actin (AlexaFluor488 Phalloidin, Invitrogen) and nuclei (NucSpot 650, Biotium). The expected region on the forward scatter versus side scatter (FSC-SSC) plot correlated well to regions of varied size and similar fluorescence (nuclei) as well as regions with a linear size-fluorescence relationship (actin). Flow cytometry results were recorded as the median fluorescence per sample for a minimum of 1,000 cells. Results are displayed normalized to the control (glass) median, with representative plots of the fluorescence versus count.

### 2.4 Microscopy and analysis

#### 2.4.1 Confocal microscopy

All samples were prepared on glass bottom plates (#1.5H cover glass, Cellvis) unless otherwise specified. Images were collected via Olympus Fluoview 3000 using UPLXAPO-4X, UPLXAPO-10X, and UPLXAPO-20X objective lenses. Excitation lasers of 405-nm and 488-nm were used, with detection bandwidths of approximately 50 nm. The imaging window was defined as the x-y plane. The main window size was typically 636.4 × 636.4 µm^2^, corresponding to 1024 × 1024 pixels^2^. All images of substrates were taken as z-stacks, with the step size optimized via the native Olympus software. Samples containing live cells were kept at 37 °C and 5% CO_2_ via Tokai Hit on-stage incubator. Migration samples were imaged for 18 total hours with a 90-min time step, utilizing the z-drift compensation (ZDC) feature and the multi-area time lapse (MATL) feature. Track length for migrating cells averaged 16.8 h for the BTM and 16.1 h for the PTM, a difference which is not statistically significant (p > 0.05). More than 80% of all included tracks were recorded for ≥ 15 h, with a minimum track length of 9 h. Imaging regions were selected that contained cells which did not physically interact with one another or with the sample edge. All visible cells meeting these conditions were imaged in each sample, with the same magnification and step size.

#### 2.4.2 Image post-processing

Microscopy images (.oir) were opened via the Bio-Formats importer in FIJI (Schindelin et al., 2012). All processing described below was subjected to manual review and adjustment where necessary. Cell viability counts were taken via the Find Maxima function (prominence >1,200, remove outlier < 2 pixels). To obtain cell shapes, image volumes were projected onto a single x-y plane (Z Project, maximum intensity in FIJI) and binarized in the actin channel. The 2D cell shape outlines were connected where necessary and filled in to create solid binary objects. Cell perimeter (P) and area (A) were measured in MATLAB (R2024b) with the RegionProps function (The MathWorks Inc., 2024). Circularity was calculated as:

$$Circularity=\frac{4\pi A}{P^{2}}$$

Where Circularity = 1 for a perfect circle. Cells represented in the morphological study come from three unique experiments, with at least three sample replicates per experiment. Due to the isotropic nature of both substrates, analysis in 2D is sufficient to examine our cellular properties of interest, and no unique information will be lost in the arbitrary third dimension. Substrate pore size distribution was measured on z-stacks which were resliced to achieve cubic voxels. Resultant images were binarized, despeckled, and outliers were removed (bright and dark, <5 µm). Final z-stacks were analyzed via PoreSpy software in Anaconda Online (Gostick et al., 2019). Migration analysis was performed on maximum intensity z-projections of the nuclei channel (2D), manually or with the TrackMate plug-in where applicable (Tinevez et al., 2017; Ershov et al., 2022). Analysis of migration in 2D is justified due to the isotropic nature of both substrates and the lack of any imposed gradient which could bias migratory direction, similar to existing studies (Ray et al., 2017; Li et al., 2025).

### 2.5 Materials characterization

Samples for characterization were washed in water and dried. Scanning electron microscopy (SEM, FEI Magellan 400 XHR SEM) was performed on samples sputter-coated with 6 nm of iridium at a 6 mm working distance and 10 kV excitation voltage. Energy dispersive x-ray spectroscopy (EDS) was performed with an attached Oxford Ultim detector. Peak identification and weight percent calculations were completed in Aztec. Three EDS detection points were measured per individual sample to confirm sample homogeneity. Final reported analysis was measured on three distinct samples from different batches, in line with existing work (De Vega et al., 2024). Fourier Transform Infrared Spectroscopy (FTIR) was performed on a Thermo Scientific Nicolet iS5 with iD5 ATR attachment (attenuated total reflectance). Spectra were baseline-corrected and then normalized using the OMNIC native Normalize function for easier spectra comparison.

### 2.6 Statistical methods

Statistical tests were performed in OriginPro (OriginLab, Version 2025) and Microsoft Excel (Data Analysis ToolPak). Statistical significance was considered when p < 0.05. Data was assessed for normality prior to significance testing (Shapiro-Wilk test, p < 0.05). All cell viability and phenotype controls were normally distributed and thus compared using two sample t-tests, with the assumption of equal (viability) or unequal (phenotype controls) variance as defined by the F-test of equality for variances. The remaining comparisons were made with the Mann-Whitney U test. Specific information is available in the respective figure captions. EDS data is presented as mean ± standard deviation.

## 3 Results

### 3.1 Substrate characterization

Representative SEM images of a BTM and a PTM sample are shown in Figure 2 as visual demonstration of the differences in pore morphology between the two substrates. The PTM has pores with highly variable diameter and internal curvature, due to the small pore throats which connect the spherical cavities (Figure 2A). In contrast, BTMs have continuous pore networks with saddle-like (negative Gaussian) curvatures at all internal surfaces (Figure 2B). The pore size distributions of representative BTM and PTM samples had comparable means (Supplementary Figure S1A), in line with previous analysis of these materials (McDevitt et al., 2019). This analysis on representative substrates demonstrates the similarity in average pore size between the two materials, which is the primary characteristic of interest pertaining to our downstream analysis of individual cell interactions with these substrates. The slight differences in the shape of the distributions is expected. The PTM is formed from relatively homogeneous spheres, while the BTM relies on the thermodynamic process of bijel formation. Previous reports have shown that, for the system utilized herein, bijels with pore sizes approaching 100 µm become less stable and more variable (Herzig et al., 2007), thought to arise from gravitational breakdown of the structure at large pore size due to the finite mechanical strength of a particle monolayer (Lee and Mohraz, 2010; Witt et al., 2013).

The chemical composition of the substrates was analyzed via EDS and FTIR-ATR. The BTM and PTM are made from the same monomer precursor solution, are subjected to the same polymerization steps, and experience nearly identical post-processing steps; thus we expect all bulk properties resulting from synthesis and processing to be the same. Characterization of these materials therefore emphasizes confirming that the one differing step, the removal of silica nanoparticles via HF on BTMs, does not result in any meaningful differences between the two materials. Successful removal of the silica particles has previously been confirmed (Lee and Mohraz, 2010), which is re-affirmed here by a lack of silicon signal in EDS. There is also no measurable fluorine signal, supporting the successful washing of all residual HF. The full EDS results are presented as average weight percents ± standard deviation: BTM: C = 65.4 ± 0.3%, O = 34.6 ± 0.3%, Na = 0.1 ± 0.1%, and PTM: C = 66.6 ± 0.3%, O = 33.3 ± 0.3%, Na = 0.1 ± 0.2%. These results (<2% difference) do not suggest any significant differences between the chemical makeup of the two samples, especially in light of known limitations of EDS on non-flat surfaces (Della Bona et al., 2007; Newbury and Ritchie, 2014; Miculescu et al., 2020; Shirley and Jarochowska, 2022). The carbon and oxygen can be attributed to the main PEGDA polymer, while the residual sodium content can be attributed to the Rhodamine B degradation agent. The presence of this amount of sodium would not be expected to influence cells, which is supported by the viability results described below. FTIR-ATR analysis (Supplementary Figure S1B) showed expected and matching peak locations in the C-H, C-O, C=C, C=O, and O-H regions for both samples. Differences in intensity, particularly in the fingerprint region, are believed to arise from slight differences in contact quality due to the porous nature of the materials.

Cell viability measurements for cells cultured in a substrate-containing environment, but without direct contact (transwell), showed >90% viability across all samples and replicates, with no statistically significant differences between any two groups (N = 3, p > 0.05). This demonstrates the lack of any significant leaching molecules which may influence cell behavior. Notably, the lack of significant difference in this cytotoxicity study, in conjunction with a 0% fluorine EDS signal, further support that the HF etching step, which is the only treatment step that differs between the two substrates, does not introduce any discrepancy in properties between the two substrates. Cells seeded within substrates were live/dead stained to qualitatively confirm that cells also survive within the substrates. Representative images are shown in Supplementary Figure S2. These images also demonstrate that rounder cells found in these substrates are living, an important point when analyzing cells for their morphology and migration in later sections. Cell adhesion to the substrates is believed to arise from non-specific serum protein adsorption, as seen in existing work (Sun et al., 2014; Swartzlander et al., 2014). Because of the identical precursor chemistries, parallel preparation procedures, and existing characterization data, the composition and quantity of adsorbed proteins are expected to be comparable across both materials. Dead cells were generally not visible within either substrate, despite being visible in the transwell study which was performed with the same procedure, suggesting that cells which do not adhere or die fall out or are washed away during processing.

### 3.2 Cell shape and size *in situ*

**FIGURE 3:**
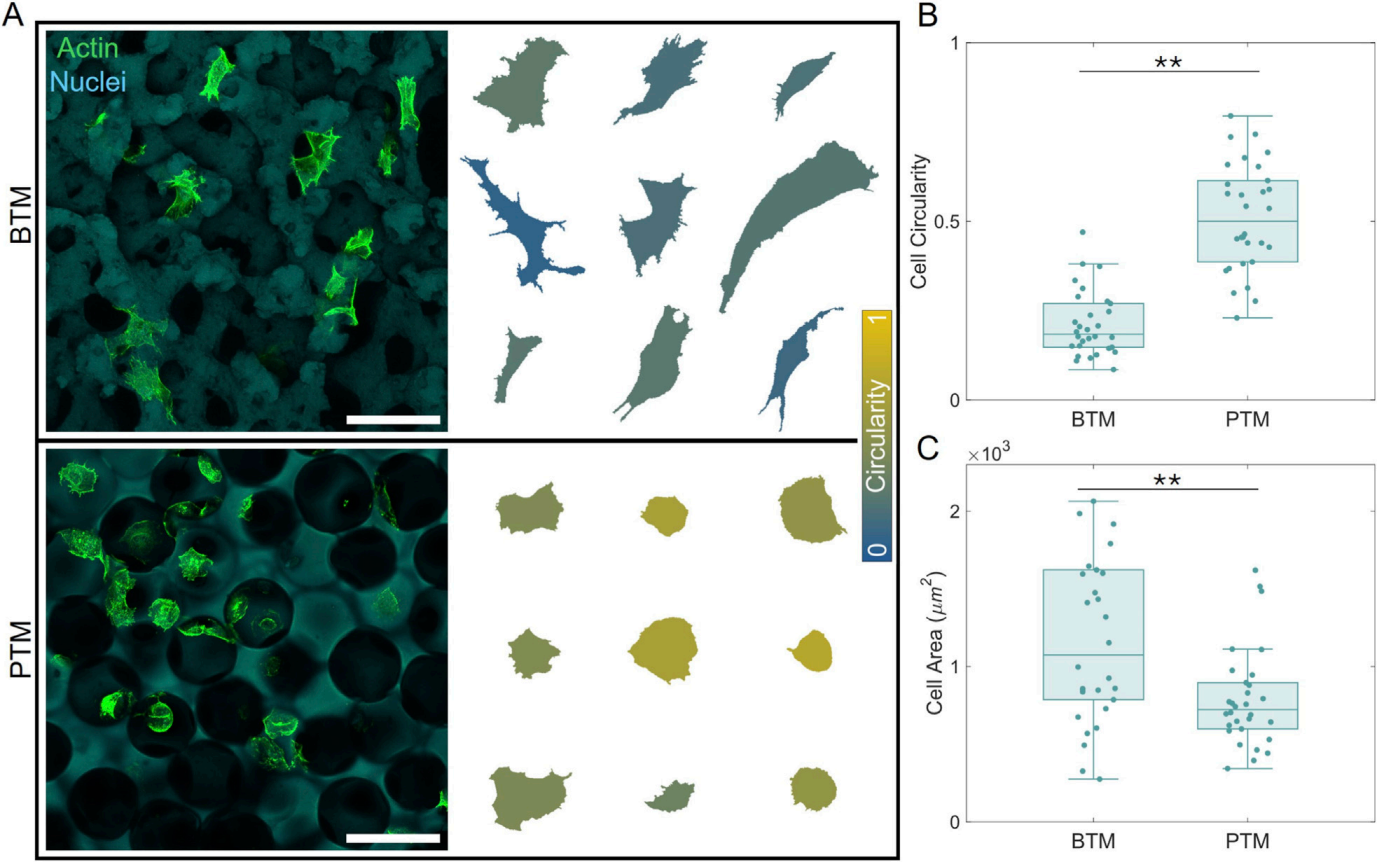
Substrate morphology influences cell shape metrics. **(A)** Confocal microscopy images of cells within each material (left) alongside enlarged, to relative scale, depictions of the resultant cell shapes (right), colorized according to their circularity. **(B)** Circularity and **(C)** cell area quantifications reveal significant differences between cells in BTMs versus PTMs (N = 30, **p < 0.01). Scale bar, 100 µm.

The shape of NHDFs within the substrates are shown in Figure 3. Representative maximum intensity z-projections are displayed alongside a set of the analyzed cell shapes in each sample, colorized according to their circularity. Cells within the PTM appear more circular than cells in the BTM which appear to elongate and adapt highly anisotropic shapes throughout the material (Figure 3A). Fibroblasts were considered for shape and area analysis only if they were at least a few microns past the cut surface of the material to minimize wall effects. Quantification of the circularity (Figure 3B) and area (Figure 3C) of the fibroblasts revealed statistically significant lower circularity and higher area for cells interacting with the BTM (N = 30, **p < 0.01).

### 3.3 *In situ* migration

**FIGURE 4:**
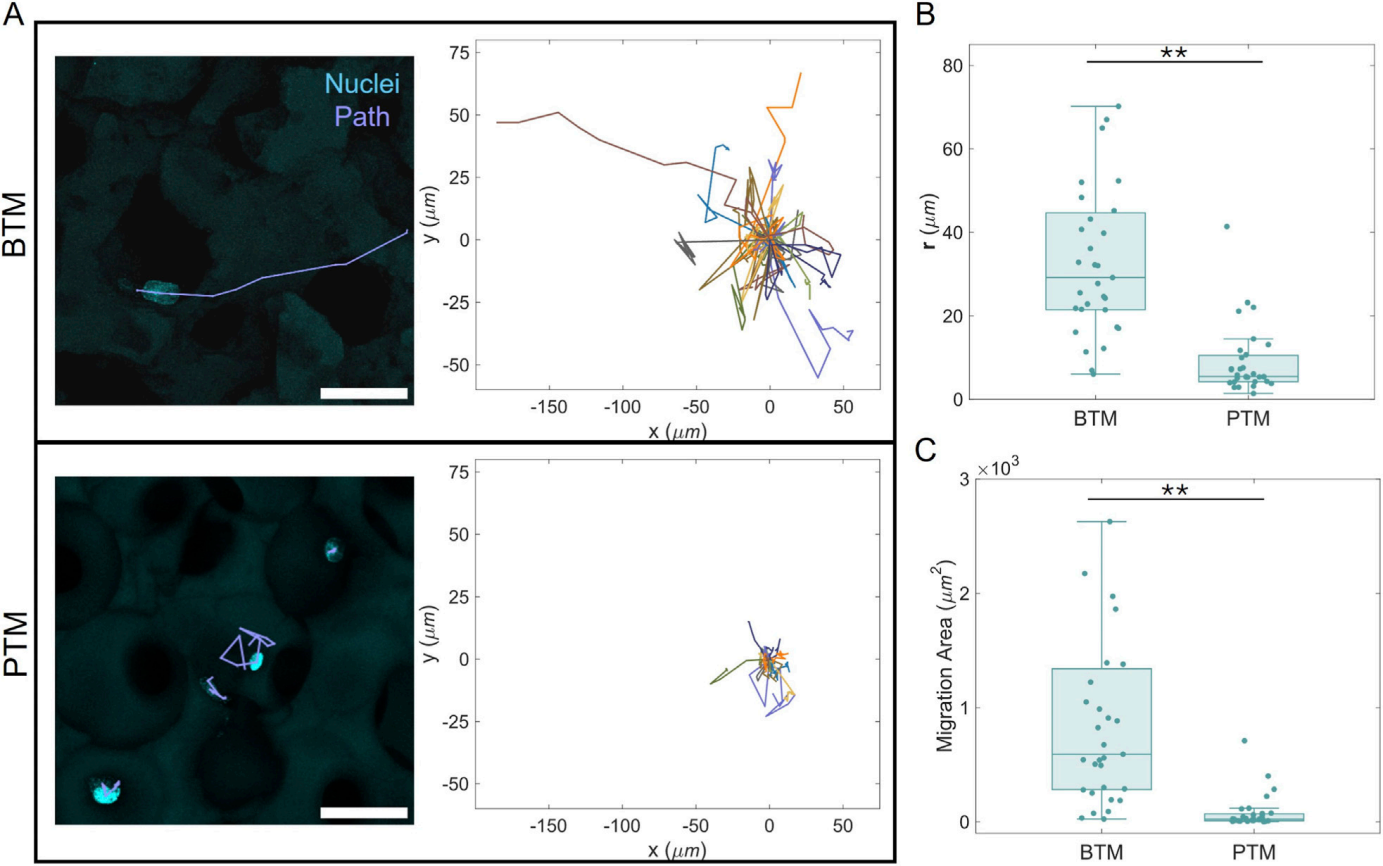
Migration of fibroblasts in porous substrates. **(A)** Exemplary paths in the substrates are shown (left) alongside all migration paths (right) for each substrate. Quantification metrics of **(B)** radius from origin (r) and **(C)** total migration area reveal significant differences in migration behavior (N = 31, **p < 0.01). Outliers for the BTM in **(B)** at 191 µm and **(C)** at 9,486 μm^2^ are not shown. Scale bar, 50 µm.

The migratory paths of measured cells are included in Figure 4A, with example cell paths traced in microscopy images (left) and all paths displayed (right), adjusted such that the origin of each path is at (0,0). The quantification metrics of both radius from origin (Figure 4B) and migration area (Figure 4C) showed statistically significant differences (N = 31, **p < 0.01) between the two porous substrates. Cells in BTMs tended to migrate across greater overall areas and further from their initial locations. Cells in PTMs tended to move much less overall, and those that did migrate tended to remain in their initial spherical pore (Figure 4A, bottom left). Example paths and quantified metrics for fibroblasts on glass are provided in the supplement (Supplementary Figure S3). The analysis of cells on glass is included as demonstration of recognizable and expected cell behavior, but is not intended to provide an absolute comparison to the 3D porous substrates, given previously established differences in cell behavior on 2D versus 3D constructs (Li and Kilian, 2015; Duval et al., 2017).

### 3.4 Cell phenotype

**FIGURE 5:**
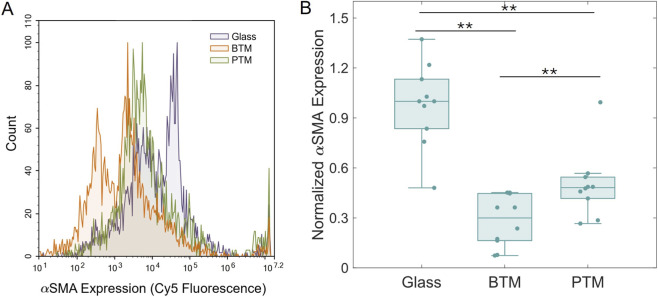
Inflammatory marker expression of fibroblasts in porous substrates. **(A)** A representative histogram showing the fluorescence versus count spectrum of one sample for each substrate type. **(B)** Cumulative results for all samples showing statistically different expression between each pair of samples (N = 10, **p < 0.01).

Phenotype of NHDFs was determined by staining for the commonly utilized fibroblast-to-myofibroblast marker αSMA, quantified via flow cytometry (Negmadjanov et al., 2015; Witherel et al., 2019; D’Urso and Kurniawan, 2020). Fibroblasts contribute significantly to the foreign body response via excess extracellular matrix deposition. We therefore use αSMA as a marker of fibroblast activation and pro-fibrotic remodeling, which is closely associated with sustained, downstream inflammatory signaling. The utility of this experiment was confirmed by testing intentionally induced (via TGF-β) and uninduced cells, which revealed significantly higher αSMA expression in the induced samples (N = 4, **p < 0.01) (Supplementary Figure S4). Results for cells on the BTM and PTM substrates revealed higher αSMA expression for cells in the PTM (N = 10, **p < 0.01) compared to the BTM (Figure 5). Both the BTM and PTM also showed statistically significant lower αSMA expression than the cells on glass, which can be attributed to the well-established relationship between stiffness and fibroblast phenotype (Shinde et al., 2017; Wang et al., 2021; Caracena et al., 2022).

## 4 Discussion

Fibroblasts were seeded in bijel-templated materials (BTMs) and particle-templated materials (PTMs) and examined after 48 h. This report marks the first *in vitro* analysis of cell behavior and phenotype in these uniquely porous materials with characteristic features at 80 µm. The results presented herein reveal that with matched material composition and comparable pore size, BTM and PTM architecture alone meaningfully influence cell shape, migration, and phenotype. The equivalence in chemistry and processing conditions used here suggests that pore architecture is the primary factor driving the observed differences. This work, then, is also the first to experimentally validate previous computational results, which predicted significant differences between cell behavior within BTMs and PTMs, driven by their disparate pore landscapes.

Fibroblasts interacting with the continuous pore structure and negative Gaussian curvature of a BTM have less circular shapes and larger areas than those interacting with the spherically interconnected pores of the PTM. This result suggests that fibroblasts cannot easily adhere and spread along the PTM pore curvature. The cell shape difference is in agreement with our existing computational predictions that cells within BTMs would take on more complex, anisotropic shapes than those in PTMs (Gonthier et al., 2023). In addition, despite the fact that the characteristic feature size of these scaffolds was specifically chosen to preclude a size exclusion effect, fibroblasts in the PTM structure appear to remain in one spherical pore. Geometrically, a fibroblast and its nucleus can easily fit through the PTM pore throats. Their tendency not to, then, suggests that the high local surface curvature at the pore throats is itself a deterrent to migration. This is further supported by specific migratory paths of fibroblasts within the PTM that do appear motile. Despite constant motion, these cells only traverse the region of their spherical pocket between the interconnects, seemingly moving away from the pore throats if they get too close (Figure 4A, bottom left). This behavior is very well aligned with the previously reported computational modeling prediction by our group, which showed that cells which locomote via actin-ratcheting respond to the high pore throat curvature by retreating back into their initial sphere (Gonthier et al., 2023). In contrast, the uniform curvature of the BTM does not appear to significantly impede migration. Fibroblasts in the BTM have a variety of apparent motilities, consistent with the heterogeneity of a general population (Parker et al., 2023), with the ability to traverse much larger distances beyond that of a single pore region. These changes to the physical behavior of fibroblasts are, based on our results, correlated to the measured difference in inflammatory expression. The curvature-motivated confinement of fibroblasts within the PTM results in higher inflammatory expression measured by αSMA, in comparison to the openly passable and continuous pore structure of the BTM.

In light of this first experimental evidence that fibroblasts experience a direct, measurably different effect from BTM and PTM pore structures, we propose three potential mechanisms by which their pore morphology contributes to the overall fibrotic response previously seen *in vivo*. First, that the concave, spherical pores of the PTM not only confine fibroblasts as demonstrated by their reduced spread and minimal migration, but also that these traits are indicative of increased cellular tension (Werner et al., 2017; Yang et al., 2022), which is associated with higher αSMA expression (Shinde et al., 2017). Second, the ability of fibroblasts to migrate within the BTM allows cells which were recruited in response to wound formation to traverse or exit the material freely. This is in contrast to the PTM, whose migration-limiting structure may entrap fibrotic cells, prolonging or worsening the local effect of their response. Lastly, that the apparent anti-inflammatory surface curvature of the BTM helps prevent terminally fibrotic myofibroblasts, which are central drivers of excessive fibrosis (Hannan et al., 2018).

The straightforward analyses of cell characteristics induced by porous biomaterials presented here provide a first look at how these distinct structures directly influence the behavior of one cell type involved in the complex foreign body response cascade. These results, further supported by their matching computational predictions, prompt numerous opportunities for further exploration, particularly into the theories presented above that connect the observed fibroblast shape and migration patterns with the overall foreign body response seen previously. Future experiments focusing on the mechanistic pathways involved in the observed fibroblast response are also warranted, including the roles of YAP/TAZ nuclear translocation, integrin β1 expression, or myosin light chain phosphorylation, all of which are implicated in myofibroblast activation and cellular mechanotransduction (Thannickal et al., 2003; Leask, 2013; Liu et al., 2015; Lin et al., 2016; Cheng et al., 2023). In addition, the direct, measurable difference in cell characteristics seen in fibroblasts suggests potential direct effects of BTM morphology on other cell types involved in the FBR, as well as paracrine signaling relationships downstream of fibroblasts. Answering these questions, informed by the existing data at the *in vivo* level and with the support of computational studies, will advance our understanding of fundamental cell-biomaterial interactions and pave the way for the design of future implantable biomaterials with inherent anti-fibrotic effects.

## 5 Conclusion

Fibroblast responses to the morphological properties of porous biomaterials are presented. Substrates templated from bijels (BTMs) have uniform and continuous pore morphologies, which positively influence the inflammatory response. BTM-seeded fibroblasts are less circular, more migratory, and less inflamed than fibroblasts seeded into substrates with non-uniform pore structures. These results suggest a direct relationship between physical characteristics induced by the BTM’s internal structure and fibroblast motility and phenotype, further supported by existing computational predictions. Continuing work should focus on the potential direct effects on other involved cell types, the stability of these results in the presence of chemokine factors found in wound-healing environments, discernment of the mechanisms responsible for the characteristics reported, and the potential combined contributions at other length scales, such as nanoscale roughness. A comprehensive understanding of this unique cell-biomaterial interaction would provide great utility in the future design of implantable biomaterials.

## Data Availability

The raw data supporting the conclusions of this article will be made available by the authors, without undue reservation.
