# Supplementary material for: Pore morphology of bijel-templated materials promotes migration and downregulates αSMA expression in human fibroblasts
Source: Front Bioeng Biotechnol. 2025 Dec 16;13:1709302. doi: 10.3389/fbioe.2025.1709302 (PMC12748194; doi:10.3389/fbioe.2025.1709302)
Supplement: Supplementary file 1 [file DataSheet1.docx]

Supplementary Material for Pore Morphology of Bijel-Templated Materials Promotes Migration and Downregulates αSMA Expression in Human Fibroblasts

Alyse R. Gonthier^a^, Elliot L. Botvinick^bcde*^, Ali Mohraz^af*^

Emails: agonthie@uci.edu, ebotvini@uci.edu, mohraz@uci.edu

*corresponding authors: Ali Mohraz – Email: mohraz@uci.edu, Phone: (949) 824-2028, Fax: (949) 824-2541; Elliot Botvinick – Email: ebotvini@uci.edu, Phone: (949) 824-9613, Fax: (949) 824-8413; Address: Samueli School of Engineering, University of California, Irvine, Irvine, CA 92697

a Department of Materials Science and Engineering, University of California, Irvine, CA 92697, USA

b Department of Biomedical Engineering, University of California, Irvine, CA 92697, USA

c Department of Surgery, University of California, Irvine, CA 92697, USA

d Beckman Laser Institute, University of California, Irvine, CA 92697, USA

e Edwards Lifesciences Foundation Cardiovascular Innovation and Research Center, University of California, Irvine, CA 92697, USA

f Department of Chemical and Biomolecular Engineering, University of California, Irvine, CA 92697, USA

1. Supplementary Figures


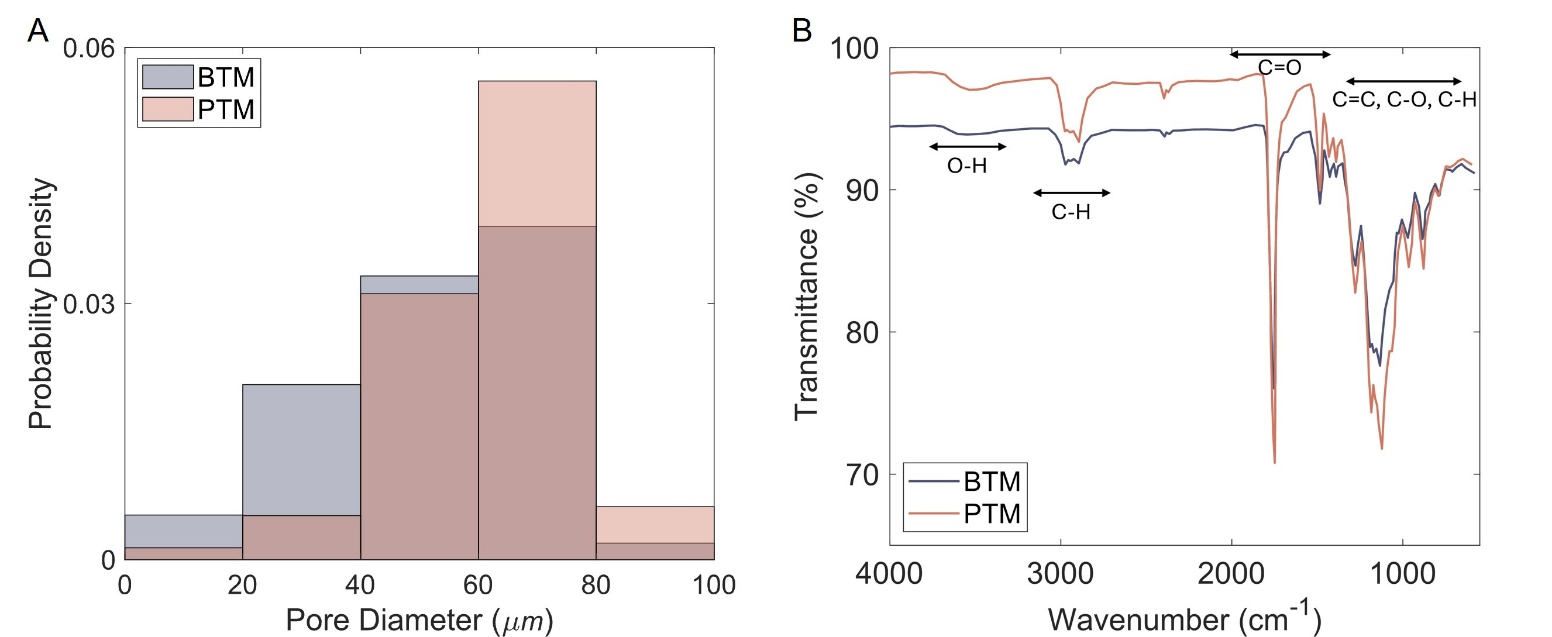


**Figure S1.** Characterization of BTM and PTM substrates. **(A)** Representative pore size distributions for each substrate, obtained from confocal microscopy images. **(B)** FTIR-ATR spectra of both samples, with relevant regions labeled.


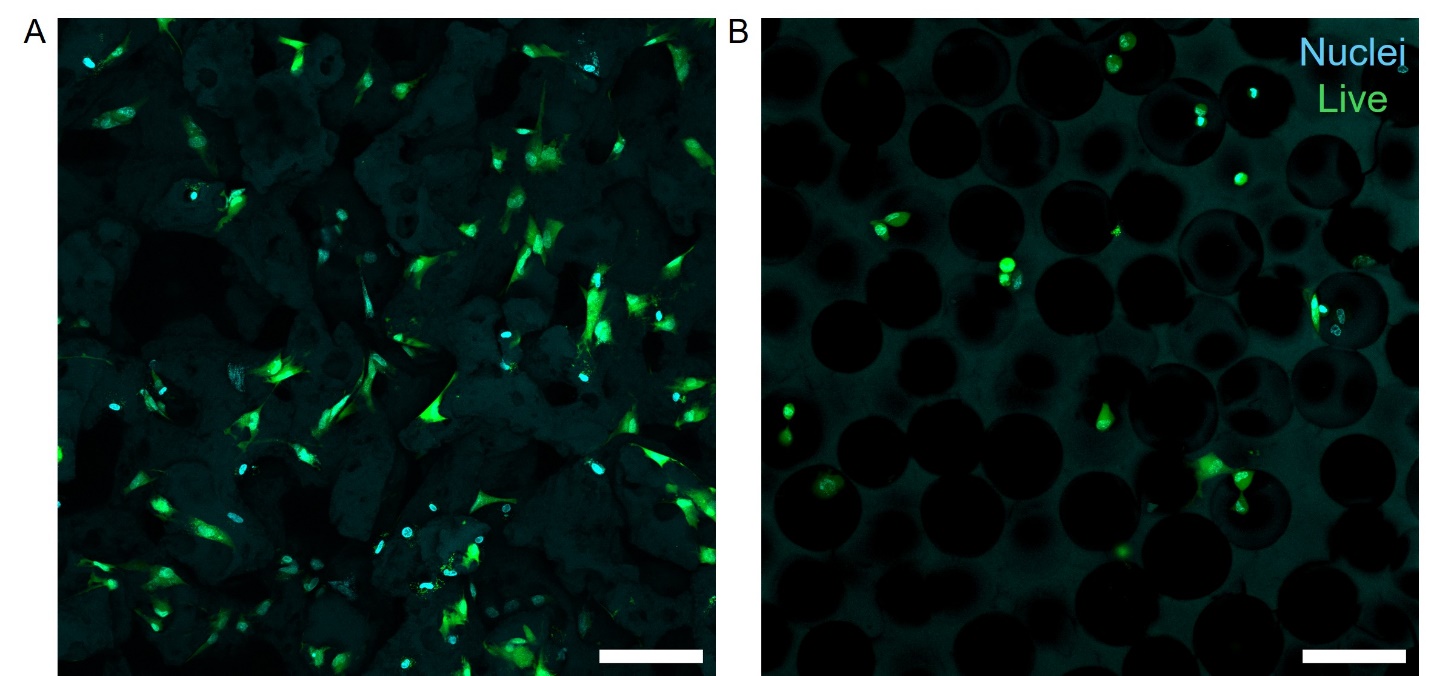


**Figure S2**. Living cells within **(A)** BTM and **(B)** PTM substrates. Live/dead stained fibroblasts are visible within each substrate via confocal z-stack maximum intensity projection. Dead cells were generally not visible within the substrates. Scale bar, 100 µm


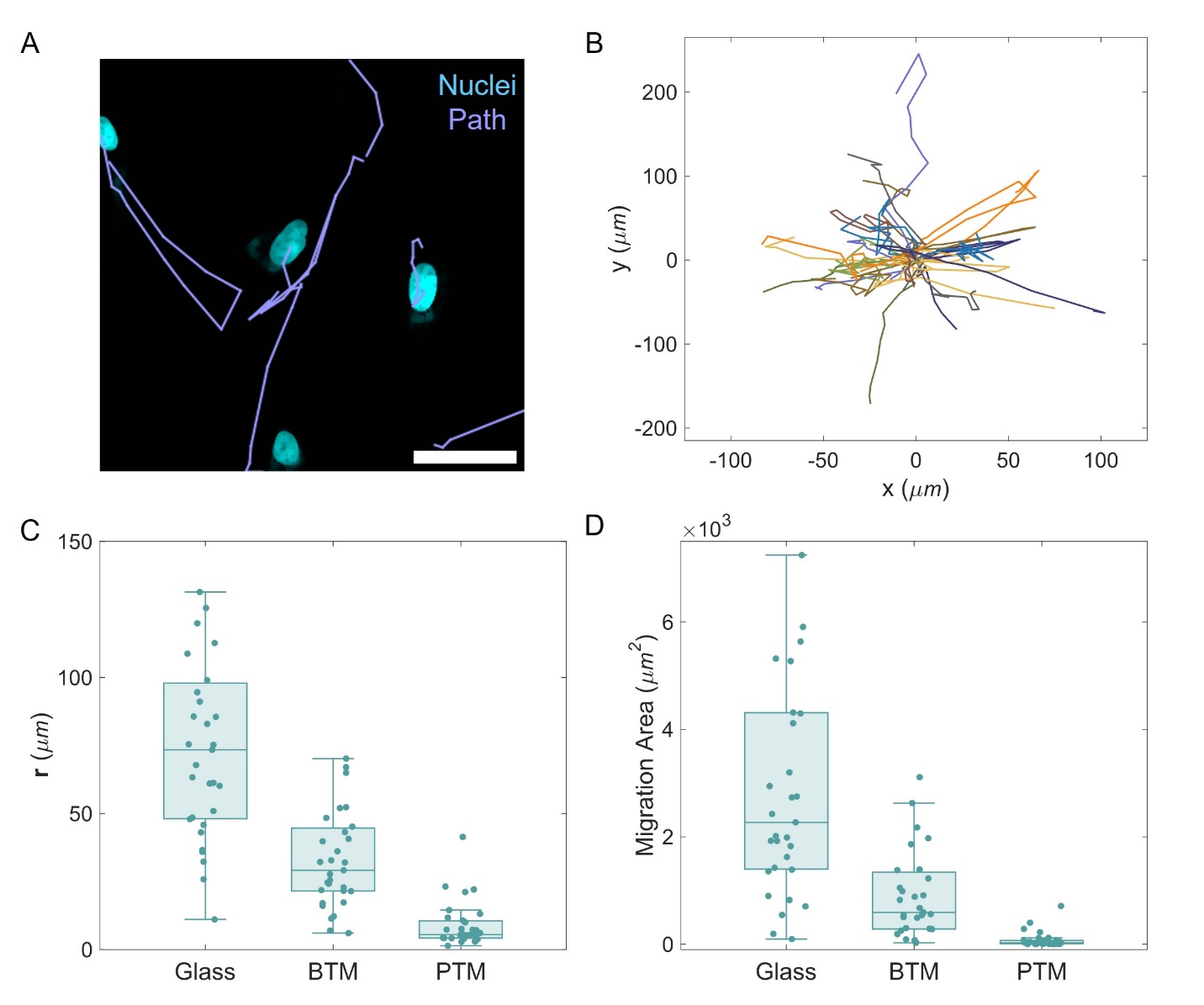


**Figure S3**. Migration of fibroblasts on glass. **(A)** Example paths are shown alongside **(B)** all migration paths (N=31). The **(C)** radius from origin (**r**) and **(D)** migration area metrics are shown in comparison to the BTM and PTM data (replicated from Figure 4). Outliers for the Glass in **(C)** at 173 µm and in **(D)** at 9998 and 10172 µm^2^, as well as for the BTM in **(C)** at 191 µm and in **(D)** at 9486 µm^2^ are not shown. Scale bar, 50 µm.


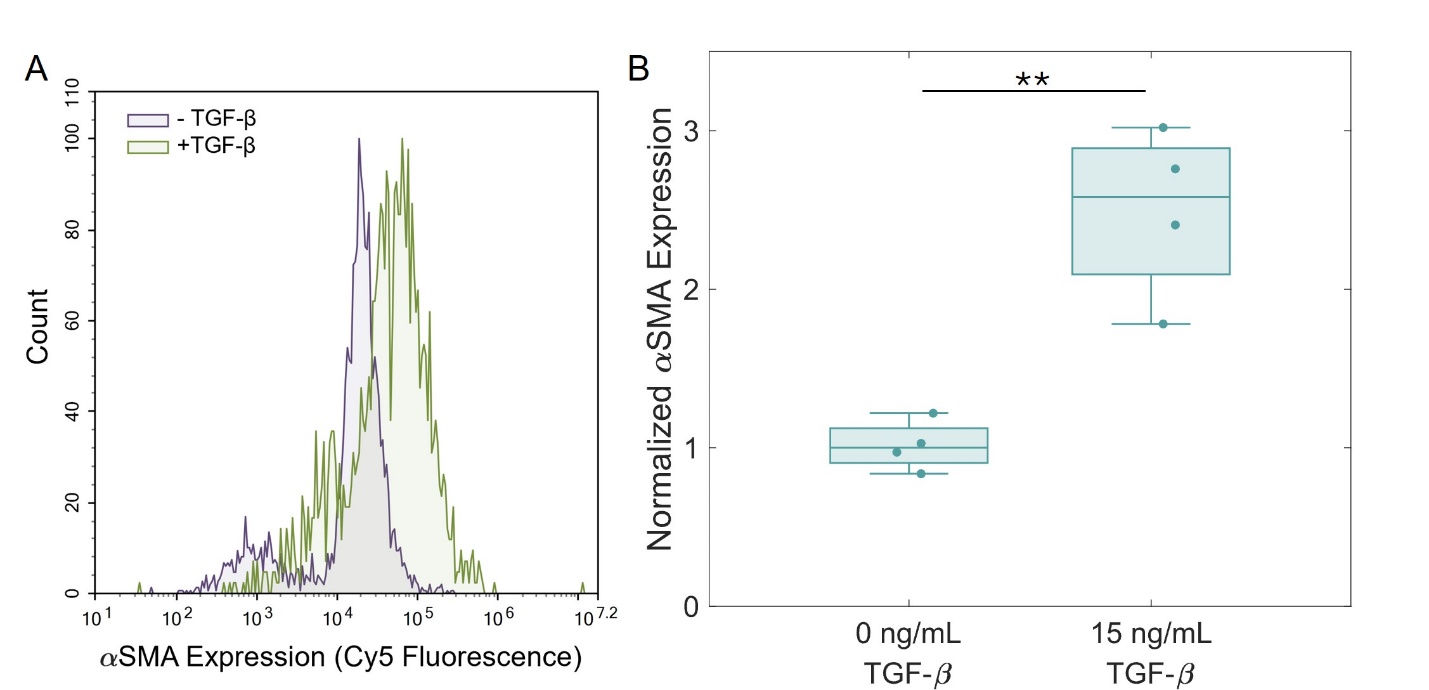


**Figure S4**. Fibroblasts are measurably inflamed after TGF-β exposure. **(A)** A representative graph comparing the fluorescence profiles of one induced (via TGF-β) and one uninduced sample. **(B)** Cumulative data comparing the inflammation states of all samples (N=4, **p < 0.01).
